# A1603P and K1617del, Mutations in β-Cardiac Myosin Heavy Chain that Cause Laing Early-Onset Distal Myopathy, Affect Secondary Structure and Filament Formation *In Vitro* and *In Vivo*

**DOI:** 10.1016/j.jmb.2018.04.006

**Published:** 2018-05-11

**Authors:** Francine Parker, Matthew Batchelor, Marcin Wolny, Ruth Hughes, Peter J. Knight, Michelle Peckham

**Affiliations:** School of Molecular and Cellular Biology, Faculty of Biological Sciences, University of Leeds, Leeds, UK; Astbury Centre for Structural Molecular Biology, University of Leeds, Leeds, UK

**Keywords:** myosin, myopathy, muscle, coiled coil, mutations, β-MHC, β-cardiac myosin heavy chain, MPD-1, Laing early-onset distal myopathy, GST, glutathione *S*-transferase, LMM, light meromyosin, EM, electron microscopy, CD, circular dichroism, WT, wild-type, MD, molecular dynamics, RMSF, root mean square fluctuation, MRE, mean residue ellipticity

## Abstract

Over 20 mutations in β-cardiac myosin heavy chain (β-MHC), expressed in cardiac and slow muscle fibers, cause Laing early-onset distal myopathy (MPD-1), a skeletal muscle myopathy. Most of these mutations are in the coiled-coil tail and commonly involve a mutation to a proline or a single-residue deletion, both of which are predicted to strongly affect the secondary structure of the coiled coil. To test this, we characterized the effects of two MPD-1 causing mutations: A1603P and K1617del *in vitro* and in cells. Both mutations affected secondary structure, decreasing the helical content of 15 heptad and light meromyosin constructs. Both mutations also severely disrupted the ability of glutathione *S*-transferase–light meromyosin fusion proteins to form minifilaments *in vitro*, as demonstrated by negative stain electron microscopy. Mutant eGFP-tagged β-MHC accumulated abnormally into the M-line of sarcomeres in cultured skeletal muscle myotubes. Incorporation of eGFP-tagged β-MHC into sarcomeres in adult rat cardiomyocytes was reduced. Molecular dynamics simulations using a composite structure of part of the coiled coil demonstrated that both mutations affected the structure, with the mutation to proline (A1603P) having a smaller effect compared to K1617del. Taken together, it seems likely that the MPD-1 mutations destabilize the coiled coil, resulting in aberrant myosin packing in thick filaments in muscle sarcomeres, providing a potential mechanism for the disease.

## Highlights

• It is unclear how mutations in the coiled coil of β-myosin heavy chain cause distal myopathy.
• A1603P and K1617del mutations reduce helicity and affect filament formation *in vitro*.
• eGFP-tagged β-myosin heavy chain abnormally accumulates at the M-line of sarcomeres in skeletal myotubes.
• Molecular dynamics simulations provide a molecular understanding for these experiments.
• Effects on structure and packing into the thick filament provide a molecular basis for the disease.

## Graphical abstract

**Figure:**
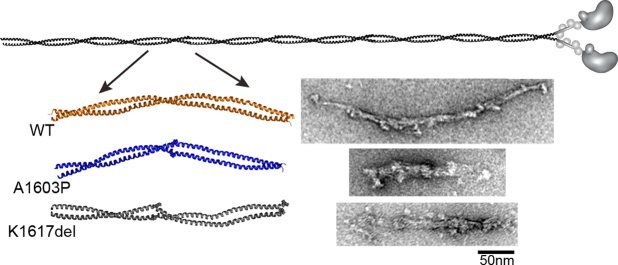

## Introduction

**Fig. 1:**
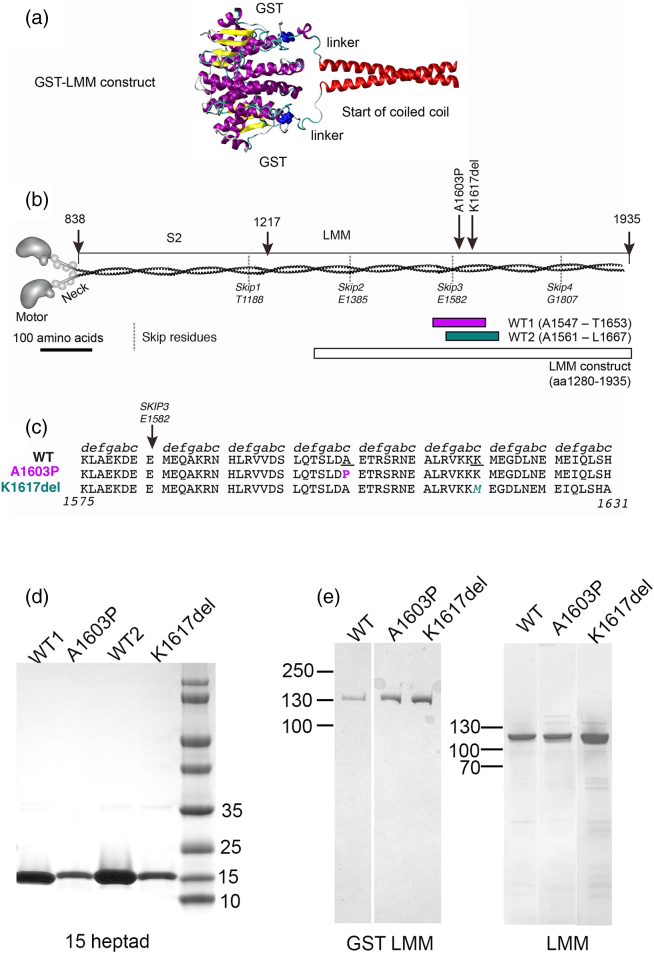
Mutant constructs. (a) A molecular model to show how GST is fused to the start of the coiled coil, in the GST–LMM constructs. Yellow: β-sheet, purple: α-helix, blue: C-terminus of GST and linker. (b) The positions of the two mutations studied here and the positions of the two 15-heptad constructs with respect to the full-length myosin, as well as the LMM construct used here, with amino acid numbers as indicated. Nominal positions of the four “skip” residues are shown. (c) Partial sequences of β-MHC, showing the WT sequence, and the mutant sequences for A1603P and K1617del underneath. The heptad assignments for WT are indicated as shown and positions of mutations as indicated. (d) SDS-PAGE gels of purified 15 heptad (15H) constructs. (e) SDS-PAGE gels of purified GST–LMM constructs and purified LMM constructs after GST has been proteolytically removed.

β-Cardiac myosin is the predominant myosin isoform expressed in slow skeletal muscle (type I) fibers as well as in the ventricles of the heart. It consists of two heavy chains (β-MHC), two essential light chains and two regulatory light chains. In common with all striated muscle myosin isoforms, the heavy chain forms the motor domain, which binds to actin and hydrolyses ATP. This is followed by the neck, which contains binding sites for each of the two light chains, and finally the coiled-coil tail. In the tail, two α-helices, one from each heavy chain, self-associate to form a parallel homo-dimer in which the two chains wind around one another in a left-handed supercoil (Fig. 1b). The coiled coil amino acid sequence contains a 7-amino-acid (heptad) quasi-repeat (***a***, *b*, *c*, ***d***, *e*, *f*, *g*), in which the amino acids in the “***a***” and “***d***” positions typically have hydrophobic side chains. This 7-residue repeat can also be considered as a 3,4 pattern. The side chains of these amino acids interact with the corresponding residues in the other chain to form a hydrophobic seam between the two helices and this stabilizes the dimer. In β-MHC, the heptad pattern is interrupted at four positions by the presence of an additional “skip” residue.

The distal region of the coiled-coil tail [light meromyosin (LMM)] self-assembles myosin into bipolar thick filaments in striated muscle. A pattern of alternating positive and negative charge arising from residues on the outside of the coiled coil (*b*, *f* and *c* residues), with a repeat of 28 residues, is required for this assembly [1]. Its periodic nature is partly responsible for the two axial staggers seen in thick filaments of 14.5 nm (98 residues) and 43.5 nm (294 residues) between molecules in the thick filament (reviewed in Ref. [2]). In the central region of the thick filament, the myosin molecules are packed in a fully overlapped anti-parallel manner, resulting in a region, 155 nm long, that lacks heads (the “bare” zone), whereas elsewhere the molecules pack in a parallel fashion. The precise way in which the LMM region of myosin tails actually packs into the mammalian thick filament backbone is unclear. However, recent cryo-electron microscopy (EM) three-dimensional image reconstructions of insect thick filaments show that the two chains of the coiled coil are associated throughout the whole tail [3] and that tails pack in a parallel fashion into the backbone forming a ribbon-like structure similar to the curved crystalline layer model originally proposed [4]. Moreover, the skip residues are important: deleting the third skip residue (E1582) prevents incorporation of a GFP-headless myosin construct into muscle sarcomeres, while skip 4 (G1807) is required for the antiparallel assembly of myosin into thick filaments in the central region of the filament [5].

While the majority of mutations in *MYH7*, the gene that codes for β-MHC, cause heart disease [6], about 30 (out of 400) mutations predominantly result in skeletal muscle disease [7], such as Laing early-onset distal myopathy (MPD-1) and myosin storage myopathy. MPD-1 is an autosomal dominant disease, which develops from age 4 up to 25 years [8]. Patients suffering from this disease initially show weakness in distal muscle groups such as finger extensors and the anterior tibial muscles, with hip abductor and rotator muscles and other selected muscle groups then becoming affected in some cases [9]. These muscle weaknesses are commonly linked to atrophy and/or depletion of type I muscle fibers [8].

Most MPD-1 mutations are concentrated into a discrete region in the coiled-coil tail, between residues 1600–1700 [6], while mutations in *MYH7* that cause heart disease are predominantly found in the motor domain (~ 60% [6]). Moreover, 60% of the MPD-1 mutations involve a substitution to a proline, with a further 20% deleting a single amino acid, while these types of mutations in the coiled-coil tail are much rarer in heart disease [6]. Both substitution of amino acids in the coiled coil to a proline and a deletion of an amino acid are expected to show strong effects on the structure of the coiled-coil tail. The cyclic nature of the proline side chain means that this residue is considered to be a “helix-breaker” [7] and it is only rarely found in coiled coils [10]. When it is found in α-helices, it introduces an abrupt bend into the helix backbone which, in a coiled coil, would be expected to disrupt the local structure, possibly unwinding the coiled coil. Similarly, deleting an amino acid disturbs the heptad repeat (*abcdefg*, 3,4), which may have an effect similar to that of introducing two “stammers,” where a stammer is 3,3,4. This might be expected to cause an over-winding of the super-coil [11]. It is unclear why mutations to proline or a single amino acid deletion in the coiled-coil tail are more strongly associated with skeletal muscle disease than with heart disease.

Studying mutations in the coiled-coil tail is challenging. The only successful approach for expressing full length β-MHC is to use cultured muscle cells, as this protein needs the correct chaperones for the motor domain to fold correctly [12]. While LMM can be expressed in *Escherichia coli*, in the absence of the bulky myosin heads, LMM forms paracrystals when it is allowed to aggregate by reducing the salt concentration [13], [14]. These paracrystals are non-physiological [15], and the relevance of these types of studies may be questionable [16]. Here we have used the novel approach of generating filaments from LMM constructs that have glutathione *S*-transferase (GST) fused to the N-terminus. The bulk of the GST dimer at the N-terminal end of the LMM dimer abrogates paracrystal formation, allowing us to investigate assembly of GST–LMM molecules into filaments. In the GST–LMM construct, the two C-termini of the GST dimer are 3.3 nm apart, on the same face of the dimer, and a linker (SDLEVLFQGPLGS) joins the C-termini of GST to the N-termini of the LMM dimer. This linker is long enough to bring the two polypeptide chains together before the beginning of the LMM coiled coil (Fig. 1a). Thus, the presence of GST is unlikely to affect LMM coiled-coil formation and the dimerizations of GST and LMM may complement each other.

Despite predictions about how mutations to a proline or deletion of an amino acid residue in the coiled coil tail might affect the structure of the coiled coil, these have not been extensively tested, and it is unclear why these mutations predominantly affect skeletal but not cardiac muscle. Therefore, we have investigated two mutations, A1603P and K1617del (Fig. 1b, c) using a combination of experimental approaches to understand how these mutations affect the normal structure and function of β-MHC, including circular dichroism (CD) to determine effects on secondary structure, EM to determine effects on GST–LMM filament formation, and imaging of GFP–β-MHC in cultured skeletal muscle myotubes and in adult rat cardiac muscle cells, to investigate effects on sarcomeric incorporation. We additionally used molecular modeling to determine the possible effects of these two mutations on the coiled coil structure *in silico*. Taken together, these data provide new insight into the effects of these mutations.

## Results

### A1603P and K1617del mutations decrease the helicity of 15 heptad and LMM constructs

**Fig. 2:**
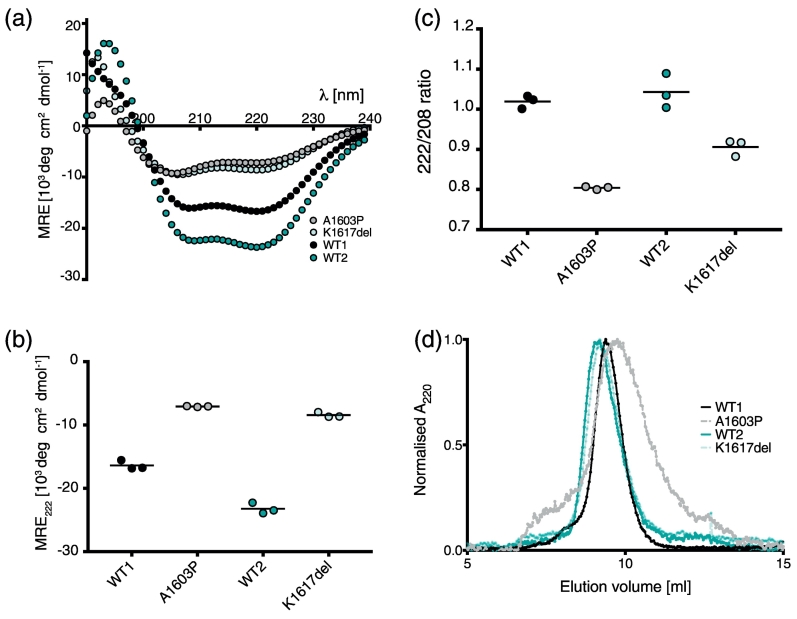
CD spectra and gel filtration for each pair of WT and mutant 15H constructs. (a) The mean spectra for each pair of WT and mutant constructs (*n* = 3) performed at a temperature of 10 °C. (b) The 222-nm value for each construct (± S.D.). (c) The 222/208 ratio for each pair of WT and mutant constructs (mean ± S.D). (d) Results from gel filtration for each of the four constructs performed at room temperature.

CD experiments showed that both mutations strongly affect the secondary structure of 15 heptad and LMM constructs. The CD spectra for the wild-type (WT) 15 heptad constructs (107 residues, Fig. 1b, d) were typical of an α-helix, with minima in the mean residue ellipticity (MRE) at ~ 208 and 222 nm (Fig. 2a). Introducing the mutations A1603P into the first WT construct (WT1) and K1617del into the second WT construct (WT2) significantly reduced the helical content (Fig. 2a, b). The ratio of mean residue ellipticity values at 208 and 222 (222/208; Fig. 2c) was above 1 for both WT constructs, indicating that they are likely to have formed coiled coil [17]. This value was somewhat reduced for K1617del and greatly reduced for A1603P, with both values below 1 suggesting that both mutations are affecting coiled-coil formation, consistent with their reduced helicity. The stronger effect of the mutation to proline on coiled-coil formation of the 15 heptad construct was also demonstrated by gel filtration experiments (Fig. 2d), in which both WT constructs and the K1617del mutant eluted in a narrow single peak in gel filtration experiments, whereas the peak for A1603P was much broader. This indicates that the proline mutation has a larger effect on secondary structure than K1617del in 15 heptad constructs. Overall, the combined CD and gel filtration data suggest that the A1603P mutation results in a largely unfolded protein, with a mixture of structures.

**Fig. 3:**
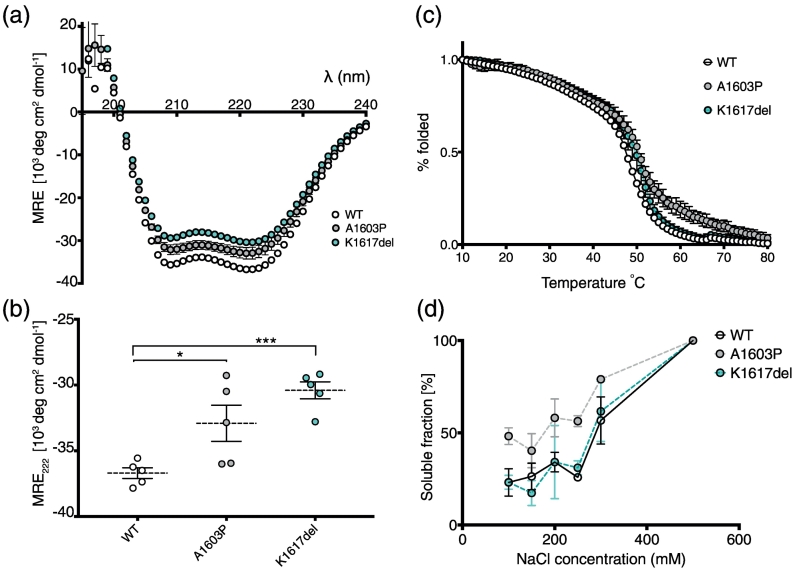
CD spectra and thermal melting curves for WT LMM and the mutant constructs. (a) CD spectra (at 10 °C) for WT and the two mutant LMM constructs. The average spectra for five experiments are shown. (b) The measured 222-nm values for WT and the two mutant LMM constructs. * and *** indicate a significant difference from WT, with *p* < 0.05 and *p* < 0.0001, respectively (*n* = 5). (c) The average thermal melting curves for each WT and mutant constructs (minimum of two experiments). The % folded values are normalized values such that fraction folded is 100% at 10 °C and 0% at 80 °C, for easier comparison. The *T*_m_ for WT is ~ 48 °C, and that for K1617del and A1603P is ~ 50 °C. (d) The solubility of WT and mutant GST–LMM constructs. Mean values ± S.E.M. are shown. *n* = 3.

Introducing A1603P or K1617del into the LMM construct (residues 1280–1935; Fig. 1b, e) also significantly reduced the helicity compared to the WT LMM construct (Fig. 3a, b). These experiments were performed in high-salt conditions (500 mM NaCl) to prevent LMM polymerization, in contrast to the experiments with the 15H constructs (performed in low-salt conditions: 50 mM NaCl). However, experiments with 15H constructs at high salt showed a similar trend to that observed at low salt (Supplementary Fig. S1). Neither of the mutations appeared to strongly affect thermal stability (Fig. 3c). A sigmoidal melting curve was obtained for each LMM construct, and the *T*_m_ for each of the constructs was ~ 49 °C.

### A1603P and K1617del mutations affect filament formation *in vitro*

Testing the solubility of GST–LMM constructs (see next paragraph) over a range of salt concentrations, demonstrated that both K1617del GST–LMM and WT GST–LMM constructs had a similar solubility profile (Fig. 3d), whereas the A1603P GST–LMM construct remained more soluble at lower salt concentrations. This indicates that A1603P GST–LMM is less able to polymerize into filamentous structures, raising the possibility that this mutation affects muscle thick filament formation by intact myosin.

**Fig. 4:**
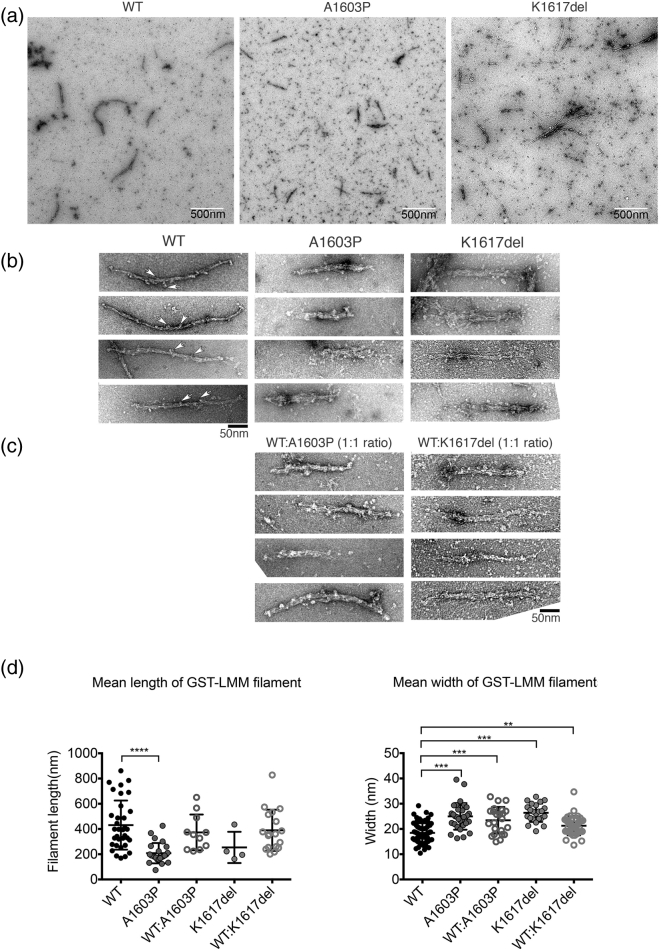
Effects of mutations on polymer formation by GST–LMM constructs that have the GST tag still attached. (a) Low-magnification electron micrographs of negatively stained WT and the two mutant LMM constructs (A1603P and K1617del). The scale bar is 500 nm. (b) Gallery of negatively stained filaments of WT and the two mutant LMM constructs (A1603P and K1617del). White arrowheads indicate GST moieties projecting from the filaments. (c) 1:1 mixtures of WT:A1603P and WT:K1617del. The scale bar for panels b and c is 50 nm. (d) Filament lengths and widths for GST–LMM constructs. Individual values are shown, together with the mean ± S.D. Length measurements were only made when the ends of the filaments were clearly identifiable and filaments were not bundled. For widths, *n* = 34 (WT), *n* = 24 (A1603P), *n* = 11 (WT:A1603P), *n* = 4 (K1617del) and *n* = 18 (WT:1617del). For lengths, *n* = 34 (WT); *n* = 24 (A1603P), *n* = 11 (wt:A1603P), *n* = 4 (K1617del) and *n* = 18 (WT:K1617del). Significant differences between WT and each of the other types of filament are indicated by asterisks: ** *p* < 0.01, *** *p* < 0.001, **** *p* << 0.001.

To investigate this further, we used EM to determine if the filaments that were formed by WT and mutant LMM, as GST-fusion proteins, were affected. We used GST–LMM, as we discovered that by leaving the GST-tag on the N-terminus of the purified LMM, it was forced to assemble into filamentous structures under low ionic strength conditions (Fig. 4). In contrast, if the GST tag is cleaved off, the resulting LMM would form paracrystals, as would purified myosin rod (consisting of LMM and subfragment 2) [18]. Thus, the presence of the GST tag disrupts the formation of paracrystals and more easily allows us to investigate the effects of mutations on filament formation.

EM of negatively stained GST–LMM filaments revealed that both mutations affect filament formation. WT GST–LMM formed filaments which had a broad range of lengths (431 ± 34 nm (mean ± S.E.M.), *n* = 34) and widths (18.4 ± 0.4 nm, *n* = 72; Fig. 4a, b). Their widths are similar to those observed for isolated thick filaments (12–18 nm [19]). A1603P GST–LMM filaments were significantly shorter (208 ± 16 nm, *n* = 24) and wider (25 ± 1 nm, *n* = 31; Fig. 4). A 50:50 molar ratio of WT and A1603P mutant GST–LMM, mixed prior to filament assembly, generated an intermediate phenotype with an average length of 390 nm and width of 22 nm (Fig. 4c). K1617del GST–LMM filaments were also wider than WT GST–LMM filaments (Fig. 4). This mutant also showed a greater propensity to form large filament bundles (Fig. 4a), and individual molecules were more apparent in the background, suggesting poorer filament formation (Fig. 4a). Approximately 70% of the EM images of K1617del filaments contained filament bundles, compared to only 19% for WT. A 50:50 molar ratio of WT and K1617del filaments had intermediate lengths and widths and a lower propensity to form bundles (Fig. 4c, d). Thus, both mutations affect filament assembly *in vitro*.

### Tail mutations affect the localization of myosin into the muscle sarcomere in cultured skeletal muscle myotubes

As both mutations affected filament formation *in vitro*, we expected that they would also affect the ability of full length myosin, tagged with eGFP for visualization, to incorporate into muscle sarcomeres in cultured skeletal muscle myotubes. In these experiments, the adenoviral constructs used to introduce eGFP–β-MHC are added to the cells (skeletal muscle myoblasts) just prior to fusion into myotubes, and thus before thick filaments and sarcomeres have begun to form. This means that the eGFP–MHC can interact with the endogenous MHC to form co-filaments, thereby testing the ability of the eGFP–MHC to incorporate into *de novo* filaments.

**Fig. 5:**
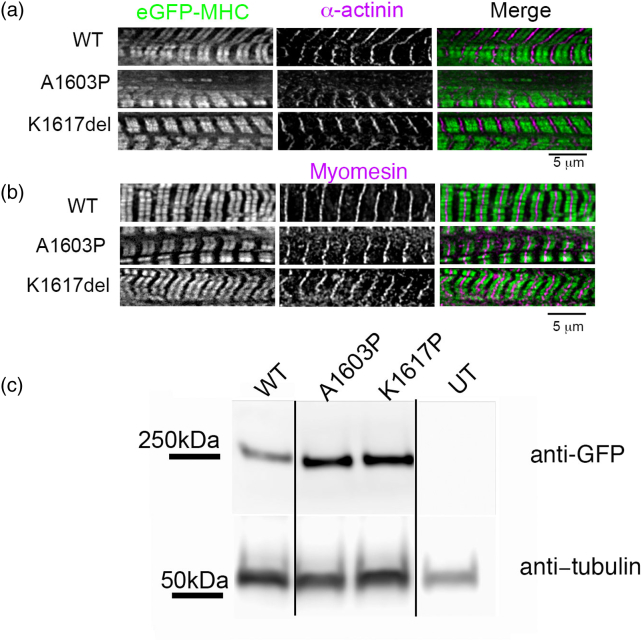
Incorporation of eGFP–MHC constructs into sarcomeres in cultured skeletal muscle myotubes formed by C2C12 cells. (a) Fluorescent images of skeletal muscle myotubes expressing eGFP–MHC constructs (green in merged image) co-stained for the Z-disc protein α-actinin (magenta in merged image). (b) Fluorescence images of skeletal muscle myotubes expressing GFP–MHC constructs (green in merged image) co-stained for the M-line protein myomesin (magenta in merged image). (c) Western blots for untransfected skeletal muscle myotubes (UT) and myotubes expressing WT and mutant eGFP–MHC constructs probed with anti-GFP. Gel lanes are approximately equally loaded, as shown by the blot probed with anti-α-tubulin (as a loading control).

Both WT and mutant eGFP–β-MHC constructs incorporated into *de novo* filaments in developing skeletal muscle myotubes (Fig. 5a). The coiled-coil tails are expected to pack in an anti-parallel fashion in the central region (bare zone) of the thick filament, leaving a small region (~ 0.16 μm) that is devoid of motor domains. As the eGFP is on the motor domain, two fluorescent stripes per sarcomere are thus expected, with low intensity in the bare zone. Both WT and mutant eGFP–β-MHC constructs appeared to incorporate with this general pattern (Fig. 5a). Staining patterns for α-actinin, a Z-disc protein, were similar for WT and mutant eGFP–β-MHC constructs (Fig. 5a). Staining patterns for myomesin, a myosin binding protein present only at the M-line, were also similar for WT and the two mutant eGFP–β-MHC constructs (Fig. 5b). Thus, although both mutations lie within a region that contains the binding sites for myomesin, (residues 1503–1671 [20], [21]), they do not appear to affect recruitment of myomesin to the M-line. Western blots show that the WT and mutant isoforms expressed are the correct size (Fig. 5c).

**Fig. 6:**
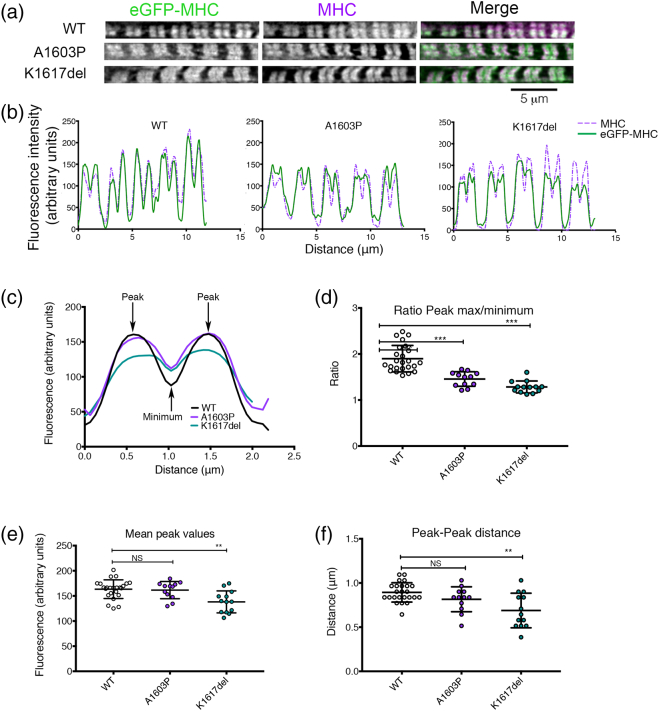
Mutant isoforms of eGFP–MHC affect incorporation into muscle sarcomeres in cultured skeletal muscle myotubes formed by C2C12 cells. (a) Images of individual myofibrils within cultured skeletal muscle myotubes expressing eGFP–MHC constructs as shown and co-stained for skeletal myosin using the A4.1025 antibody. (b) Example line profiles for MHC (magenta) and eGFP–MHC (green) across five sarcomeres from the images shown in panel a. (c) Mean fluorescence intensity profiles for eGFP–MHC organization across a single sarcomere, for WT and each mutant. The mean plot profile was calculated from measurements of at least 12 myofibrils (with 2–5 sarcomeres measured individually per myofibril) from different myotubes (*n* = 24, WT; *n* = 12, A1603P; *n* = 13, K1617del). Positions of the peak intensity values and the minimum intensity value (at the M-line) are indicated for WT eGFP–MHC. (d) The ratio between the mean peak intensity values (as in panel c) and the mean minimum intensity value at the M-line. (e) The mean peak values (averaged for both peaks, either side of the M-line) for WT eGFP–MHC and each of the mutants. (f) The mean peak–peak distance between peak maxima either side of the M-line. In panels d–f, each data point shows the average value for a single myofibril. The mean ± S.D. for each mutant is also shown overlaid as bars. Two-tailed *t* tests were used to compare mean values between the WT and mutant constructs (* *p* < 0.05, ** *p* < 0.001, *** *p* < 0.0001).

Analyzing the incorporation of eGFP–MHC into sarcomeres in skeletal muscle myotubes in more detail showed that the fluorescence pattern for eGFP–MHC was similar to the staining pattern obtained with an antibody to skeletal myosin (Fig. 6a). The antibody (A4.1025 antibody, for which the epitope is also in the motor domain [22]) is expected to recognize both endogenous myosin and transfected eGFP–MHC. A line scan along five sarcomeres for eGFP fluorescence and for the skeletal myosin antibody staining (using the images in Fig. 6a) demonstrates the similarity in staining pattern (Fig. 6b), especially for the WT construct. Incidentally, while Western blotting (Fig. 5c) does show variation in overall expression levels across the culture, all the analyses performed below used individual myotubes in which the expression levels, as judged by fluorescence intensity, were similar.

The fluorescence intensity profiles (obtained from averaging line scans along multiple single sarcomeres) were distinctly different for WT eGFP–MHC compared to the two mutant constructs in skeletal muscle myotubes (Fig. 6c). The ratio between the mean peak intensity values and the mean minimum intensity values at the M-line was significantly reduced for both A1603P and K1617del (Fig. 6d). This is mainly due to an increased minimum intensity value at the M-line compared to WT (Fig. 6c). This accumulation of A1603P at the M-line is consistent with the earlier observation where a GFP-LMM construct containing this mutation accumulated at the M-line in skeletal muscle myotubes and neonatal rat cardiomyocytes [23]. Interestingly, not only does K1617del accumulate at the M-line, but the mean peak intensity values for K1617del and the distance between the two peaks either side of the M-line are also significantly lower than the values measured for WT eGFP–MHC (Fig. 6e, f), suggesting that this mutant may incorporate less well throughout the muscle sarcomere. Thus, both mutations affect the packing of K1617del and A1603P into muscle sarcomeres.

### Tail mutations affect the localization of myosin into the muscle sarcomere in adult rat cardiomyocytes

We additionally explored the ability of WT and mutant eGFP–MHC isoforms to incorporate into the sarcomeres of adult rat cardiomyocytes. In contrast to skeletal muscle myotubes, eGFP–MHC has to incorporate into pre-existing, mature muscle sarcomeres in these cells. Adenoviral eGFP–β-MHC constructs were incubated with freshly isolated adult rat cardiomyocytes for 24 h, to express the eGFP constructs in the adult cardiomyocytes.

**Fig. 7:**
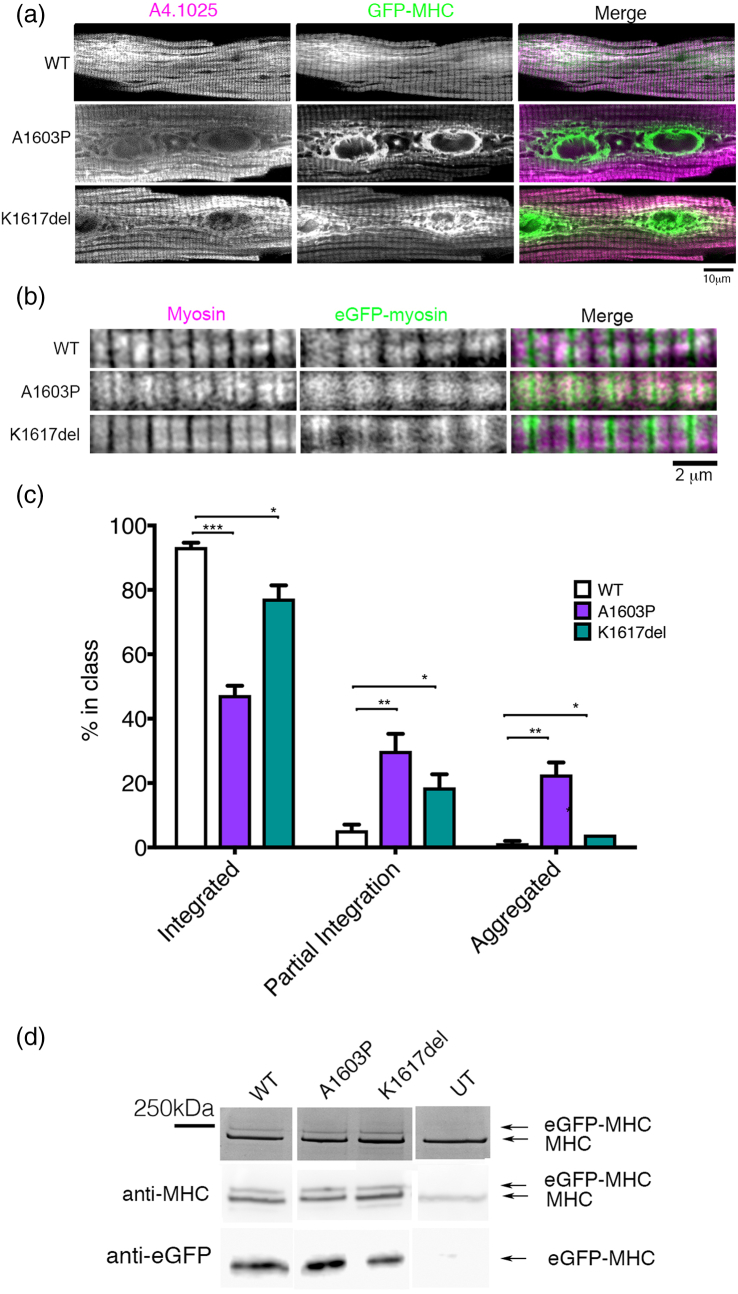
Mutations in eGFP–MHC affect incorporation into sarcomeres in adult rat cardiomyocytes. (a) Representative images of single adult rat cardiomyocytes to show the variation in incorporation of eGFP–MHC into the sarcomere and/or aggregates. Green: eGFP–MHC, magenta: anti-myosin heavy-chain antibody (anti-MHC, A4.1025). Similar MOIs (multiplicity of infection) were used for each adenovirus for each eGFP–MHC construct. (b) Typical images of a small number of sarcomeres shown at higher magnification to demonstrate how mutant eGFP–MHC isoforms can have a reduced incorporation (c) The plot shows the results of a quantitative analysis, to estimate the overall percentage of cells that either show integrated, partially integrated or aggregated eGFP–MHC in the adult cardiomyocytes for WT eGFP–MHC and each mutant. Mean values for three separate experiments are shown, with the mean ± S.D. for each construct tested (*n* = 3). Significant differences from the WT results are indicated by asterisks (* *p* < 0.05, ** *p* < 0.001, *** *p* < 0.0001). (d) SDS PAGE gel of cardiomyocytes that express the eGFP–MHC constructs or were not infected with adenovirus (UT). Two bands attributable to the endogenous MHC and the eGFP–MHC are indicated by the arrows. Western blot of the same samples for MHC (using A4.1025) is shown underneath. The two bands attributable to endogenous MHC and eGFP–MHC are indicated. The Western blot for the same samples, using anti-GFP is shown underneath.

We found that WT eGFP–MHC incorporated normally into the sarcomeres of adult rat cardiomyocytes (Fig. 7a, b) as described previously [24]. However, both eGFP–mutant MHC constructs had a reduced sarcomeric incorporation in cardiomyocytes compared to WT eGFP–MHC (Fig. 7a, b). For example, K1617del sometimes accumulated at the edges of the thick filament and did not incorporate uniformly throughout the sarcomere (Fig. 7b), while A1603P showed low levels of incorporation throughout the sarcomere. Both mutations formed aggregates of eGFP–MHC in the central region of the cell near the nuclei in some cells (Fig. 7a). To quantify these apparent changes in integration, we analyzed the numbers of cells in which myosin incorporation appeared normal, integration was partial, or myosin was aggregated (Fig. 7c). This analysis showed that these differences in myosin incorporation were significant (Fig. 7c). SDS-PAGE gel analysis together with Western blotting (to confirm the identity of the two bands in the SDS-PAGE gel) revealed that the expression levels of eGFP–MHC were similar for WT and both mutants (Fig. 7d). Thus, the mutant eGFP–MHC isoforms are poorer at incorporating into filaments in adult cardiomyocytes.

### Molecular dynamics simulations show clear structural changes for the mutants

The experimental data show that both mutations affect secondary structure and packing into thick filaments, both *in vitro* and in cells. To better understand how these mutations affect the coiled-coil structure, we used molecular dynamics (MD) simulations on a recently generated composite atomic model of a 164-residue stretch of human β-cardiac myosin coiled coil that was generated from experimental structures of short, overlapping segments of the tail [25]. This model runs from residue 1526 to 1689, and incorporates both of the mutation sites that we have studied here. We also ran MD simulations on atomic models containing the A1603P and K1617del mutations, and the results from the WT and mutated structures were compared using a similar strategy to that used earlier [5], [25]. The local distances between the two helices (*D*_com_), averaged over the course of the trajectory, the RMSD of Cα atoms in structures away from the initial structure, and the RMSD clustered structures and the root mean square fluctuation (RMSF) of Cα atoms about an averaged structure were all analyzed and compared. We also investigated the solvent accessibility of the residue side chains to test for changes in the interactions between the two helices, as well as the local heptad length and angle between adjacent heptad sections in each helix.

**Fig. 8:**
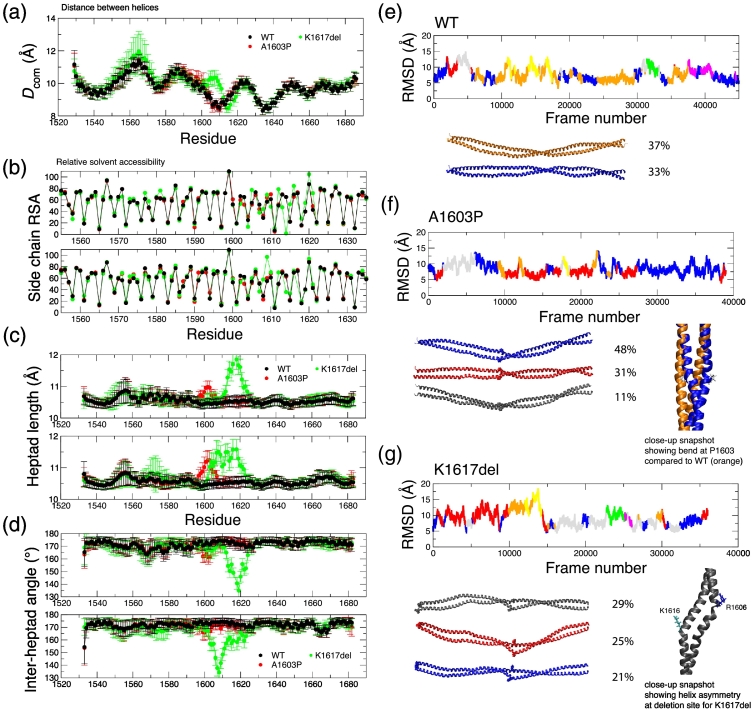
Results from MD simulation of the composite model coiled-coil and mutant models. (a) The distance between the helices (*D*_com_) as a function of residue position for WT (black), A1603P (red) and K1617del (green). Error bars represent the SD during the simulation. (b) Relative solvent accessibility (RSA) for side-chain atoms in each residue. Low values indicate solvent exclusion and thereby highlight the core residues; upper and lower plots show the RSA values for chains A and B, respectively; color scheme as per part a. Error bars are omitted for clarity. (c) Heptad length, the distance between Cα atoms in residues at positions *i* and *i* + 7, along chain A (upper) and chain B (lower). (d) Inter-heptad angle, the angle between the lines linking Cα atoms in residues at positions (*i* – 7), (*i*) and (*i*, *i* + 7), for chain A (upper) and chain B (lower). RMSD for all Cα atoms compared to the initial structure for WT (e), A1603P (f), and K1617del (g). The plots are divided by color into the different structure clusters. Example structures for the most heavily populated clusters (> 10%) are shown beneath. The position of the residue being investigated (or K1616 in the case of K1617del) is shown in spacefill. The cluster populations as a percentage of all trajectory snapshot structures are indicated. Inset for A1603P: close-up snapshot images of representative structures from the most heavily populated clusters in A1603P (blue, showing the bend at P1603) compared to WT (orange). Inset for K1617del: close-up snapshot image of the representative structure from the most heavily populated cluster showing helix asymmetry at the deletion site with bends appearing near R1606 in helix B (blue, right) and near K1616 in helix A (cyan, left).

Comparing the behavior of WT and A1603P coiled coil showed that the most marked effect of this mutation was the resulting local bend. There was a small increase in the *D*_com_ values for A1603P compared to WT just before the mutation site (Fig. 8a). The core of the coiled coil is retained, as judged by comparison of the solvent accessibility of residue side chains on both chains (Fig. 8b). The local structural change when the proline mutation is present is more clearly indicated through the increase in the heptad length (Fig. 8c) and decrease in the angle between neighboring heptads in both helices (Fig. 8d). The RMSD values are similar and give rise to seven and five RMSD-differentiated structure clusters for WT and the A1603P mutant, respectively (Fig. 8e, f). The example structure for the most commonly visited A1603P cluster (blue, 48%) shows a bend in keeping with the expectation for a proline mutation.

The K1617del mutant model exhibited several striking differences compared to WT. There are clear changes in the distance between the helices (*D*_com_) near the deletion site, with an increase in *D*_com_ for 1600 to 1612 and a small decrease up to 1620 (Fig. 8a). Changes in the solvent accessibility were also observed; V1614 plays a more core-like role where originally K1615 fulfilled the “*a*” residue role in the heptad. S1607 is also less solvent exposed than in the WT model (Fig. 8b). Both these features would appear to help to smooth the heptad pattern transition caused by the deletion resulting in a regular heptad “***a**bc**d**efg*” core residue pattern returning beyond ~ 1618. The coiled-coil model exhibits a very small loss in overall helicity (98% on average compared to 99% in WT). Close to the deletion site, there is an increase in heptad length (Fig. 8c) and a large reduction in the inter-heptad angle (Fig. 8d). Note the loss in symmetry here with the changes appearing earlier in the sequence in chain B compared to chain A, as highlighted in a close-up snapshot (Fig. 8g). The end-to-end distance of the structure (23.4 ± 0.5 nm) is on average slightly shorter compared to WT (23.9 ± 0.5 nm).

It is notable that in the WT coiled-coil simulation, several salt bridges are observed between residues close to K1617. The charge centers for pairs K1615–E1619, K1616–E1619 and K1617–D1621 within both helices are all closely positioned (< 4 Å) for over 70% of the simulation time, suggesting that these salt bridges are important for stabilizing the helical structure. Removal of K1617 results in the loss of one potential intra-helical salt bridge in each chain (using WT numbering: K1616–E1619 is no longer helix-compatible in K1617del; K1617–D1621 is also lost, but K1616–D1621 becomes possible). Simulation revealed a loss in symmetry in the salt bridge pairings; only in chain A is the K1615–E1619 salt bridge highly occupied (> 70%), while K1616–D1621 is moderately occupied in chain B (55%). Overall the average number of salt bridges (both intra- and inter-helical) in the K1617del coiled coil is 24 ± 3 compared to 26 ± 3 in the WT structure.

K1617 resides in a region of the coiled coil which, when averaged over 28 residue blocks is slightly positively charged overall. As mentioned above, the K1617del model shows some changes in the core residues compared to WT. Two charged residues (R1608 and K1615) that appear as “*a*” core residues in WT are replaced by uncharged S1607 and V1604 in K1617del, making the exposed surface of the coiled coil more positively charged in this region. It is likely therefore that, as well as changes to the structure of isolated coiled coils, the accompanying changes in the large-scale patterns of charge and hydrophobicity will affect the packing interactions with neighboring myosins within the thick filament.

Finally, we tested the possibility that the additional skip residue (E1582) might compensate for the deletion of K1617 a short distance downstream, such that a canonical coiled coil might form across the whole region. This might have some functional similarity to the loss of this skip residue, which is known to reduce myosin rod incorporation into sarcomeres [5]. We therefore built a starting model for K1617del with a canonical coiled coil throughout this region (K1617del-c), and which thus has a phase shift in the heptad repeat for all residues between 1589 and 1616 (*a* residues occupy *b* positions and so on).

The coiled-coil structure was retained during simulation of K1617del-c with some distortion around the skip residue site but barely perceptible effects on overall structure at the deletion site (Fig. S2). The RMSD was smaller and the initial structure locally more stable than for the starting model that treated the skip and deletion sites separately (Fig. S2E *cf*. Fig. 8g). The K1617del-c model as a whole also had one more salt bridge on average (25 ± 3) compared to the skip–del model simulation; the symmetry is better maintained around the K1617 deletion site resulting in both chains showing highly occupied K1615–E1619 interactions (> 70%) as well as partially occupied K1616–D1621 interactions (36% and 50% for chains A and B, respectively).

Changes in the solvent accessibility of charged residues were again observed in K1617del-c, due to the alteration in the identity of core (*a*,*d*) and exposed (*b*,*c*,*f*) residues over the range 1565–1618. For example, as with the first K1617del model, K1615 and R1608 again move to solvent exposed “*b*” positions. In addition, however, the negatively charged E1604, a “*d*” position residue in WT and other models, moves into the partially exposed “*e*” position in K1617del-c. This has the knock-on effect of removing the inter-helix E1604–R1608 salt bridges found in our WT, A1603P and first K1617del model as well as the Korkmaz *et al*. model [25]. The average end-to-end distance of the structure (23.7 ± 0.4 nm) is also closer to that of WT than K1617del. Taken together, this suggests that the deletion mutant may indeed adopt a quasi-canonical structure, as an alternative or in addition to the skip–deletion structure above, with inherent instabilities compared to WT.

## Discussion

We have explored the effect of two different MPD-1 mutations on the structure and filament forming ability of myosin constructs both *in vitro* and in cells. Both mutations had significant effects on the secondary structure of short (15 heptad, 107 residues) and long (LMM, 655 residues) coiled-coil MHC constructs, decreasing helical content, and disrupting coiled-coil formation, particularly for A1603P. Filament formation *in vitro* was also affected. A1603P GST–LMM was more soluble at low salt, and formed shorter and wider GST–LMM filaments suggesting that this mutation interferes with LMM polymerization. While the solubility profile of K1617del GST–LMM was more similar to WT, GST–LMM filaments again tended to be shorter with increased widths, and a higher tendency to aggregate together, compared to WT. These effects *in vitro* were mirrored by those seen in cultured muscle myotubes, where both mutations altered the pattern of packing of GFP-tagged β-MHC into muscle sarcomeres, with K1617del having the larger effect. Both mutations also reduced the incorporation of GFP-tagged β-MHC into pre-existing muscle sarcomeres in adult cardiomyocytes. Molecular modeling confirmed effects on the secondary structure. Overall, our finding that A1603P and K1617del mutations significantly affect the secondary structure of the coiled coil, filament formation *in vitro* and incorporation into muscle sarcomeres *in vivo* is consistent with the early onset of the disease reported in patients [26].

The strong effects of A1603P and K1617del mutations on the secondary structure of the myosin coiled coil are not surprising. However, it is interesting that these two MPD-1 mutations have stronger effects on secondary structure compared to mutations that cause hypertrophic cardiomyopathy (N1327K, E1356K and R1382W), which we investigated previously [24]. Moreover, previous studies that characterized mutations that cause myosin storage myopathies (L1793P, R1485W, E1886K and H190L) and mutations in the same residue that either causes MPD-1 (R1500P) or dilated cardiomyopathy (R1500W) did not alter the secondary structure of LMM [14], [27]. The changes to secondary structure for the two mutations tested here may arise from their positions in a fairly flexible area of the coiled coil just downstream from the third skip residue [5]. Our modeling data suggest that the deletion of a residue (K1617del) in this region is likely to affect core residue packing and stabilizing salt bridge interactions, which would be expected to reduce helicity and coiled-coil formation over longer timescales, consistent with our experimentally observed effects on secondary structure. The smaller effect of A1603P on the secondary structure of LMM compared to K1617del is consistent with our modeling results that show that while the proline mutation causes an obvious local bend in the coiled coil structure in modeling, it exhibits a less dramatic wider effect compared to the deletion mutation.

Both mutations affect filament formation *in vitro*, as demonstrated using GST–LMM filaments, and incorporation of eGFP–MHC into muscle thick filaments in skeletal muscle myotubes, with both mutants demonstrating abnormal accumulation at the M-line. Our modeling suggests that the K1617del mutation could make the exposed surface of the coiled coil more positively charged close to the deletion site, or alter the surface charge more widely (as found in the K1617del-c model). Either change would be expected to have effects on the longer range interactions between neighboring myosin tails within the thick filament, thus affecting tail packing, which is known to depend on the pattern of positive and negative charges along the length of LMM [1]. These effects would thus explain the aberrant filament formation we observed both *in vivo* and in cells. The bend in the coiled-coil introduced by the mutation to proline in A1603P demonstrated by the modeling is also expected to affect the ability of this mutant to pack into the filament.

Overall, the strong effects of these two mutations on secondary structure and filament formation *in vitro* and in cultured skeletal muscle cells help to explain why these mutations result in the phenotype reported for patients. Both A1603P and K1617del cause scoliosis of the spine, reduced respiratory capacity and weakened distal muscle [26], [28]. However, β-MHC is also expressed in cardiac muscle, and we found that both mutations reduced levels of eGFP-β MHC incorporation into sarcomeres in cultured adult rat cardiomyocytes. However, neither of these MPD-1 mutations are thought to affect cardiac muscle in patients. In our experiments, the expressed eGFP–β-MHC has to incorporate into pre-existing myofibrils in the cultured adult cardiomyocytes. Our results suggest that the mutant isoforms incorporate into sarcomeres more slowly than WT under these conditions, and they might eventually incorporate into the cardiac thick filaments over a longer time period. However, the *in vitro* cardiomyocyte culture system does not allow us to test this, as the cardiomyocytes quickly degenerate (over 2–3 days after isolation) once in culture. If this slower rate of incorporation does reflect a lower incorporation of mutant protein in cardiomyocytes in patients, this may allow for preferential incorporation of normal myosin, and possibly a reduced effect of these mutations on the muscle sarcomeres. Moreover, as the rate of myosin turnover has been measured to be higher in the heart (half-life of ~ 15 days) compared to skeletal muscle (half-life of ~ 45 days [29]), this may allow the mutant myosin that fails to incorporate to be cleared at a faster rate, while in skeletal muscle, there is an increased chance of the mutant myosin incorporating into the sarcomere, thereby generating the predominantly skeletal muscle phenotype.

The use of GST–LMM has allowed us to develop a novel approach to investigate the effect of mutations in filamentous structures *in vitro*. The presence of the dimeric GST moiety at the N-terminal end of the LMM construct does not interfere with coiled-coil formation, but prevents paracrystal formation, and thus allows us to test filament formation via negative stain EM and solubility assays using protein rapidly and economically expressed in bacteria. This approach demonstrated that both A1603P and K1617del mutations affect filament formation *in vitro*, in agreement with the effects on secondary structure, the modeling results and the effect of eGFP–MHC incorporation in cells. Using GST–LMM should be useful in future, to examine the effects of other coiled-coil mutations on filament formation.

Overall, the two mutations studied here have relatively strong effects on the secondary structure of the myosin tail in the coiled coil. We have also described a new and exciting technique to study *in vitro* filament formation of the LMM proteins and demonstrated that both A1603P and K1617del also have a strong effect on filament formation. Our finding that a mixture of WT and mutant myosins has a strong effect on filament formation is consistent with the autosomal dominant nature of this disease. Effects on the structure of the coiled coil are thus likely to affect filament formation and sarcomere incorporation, suggesting a potential mechanism for MPD-1.

## Experimental Procedures

### Generation of mutants and short constructs

The full-length cDNA used in these experiments encoded human MYH7 (P12883, Uniprot) and was cloned into the pdc315 adenoviral vector with eGFP fused to the N-terminus as previously described [24], [30]. The amino acid sequence between the end of the coding sequence for eGFP and the start of that for MYH7 is SESRACSLEACGTVDSMHHHHHHHH. For expression in mammalian cells, two point mutations were made in the full-length cDNA for the heavy chain in the pdc315 vector using the Quick Change-XL II Site Directed Mutagenesis Kit (Agilent) according to the manufacturers' instructions. Each construct was sequenced to verify that the desired mutations had been introduced and to confirm that no other mutations had been introduced.

For expression in *E. coli*, both 15 heptad (15H) and LMM constructs were expressed as GST-fusion proteins. First, WT 15H constructs (WT1 and WT2) were designed, in which the central heptad contains the mutation, in the mutant version of the construct. Both constructs were additionally designed to start and finish on an amino acid in the *d* position of the heptad repeat. The LMM construct contained residues 1280–1936. The 15H and LMM regions were amplified from the full-length cDNA using PfuUltra High Fidelity Polymerase (Agilent) with primers containing BamHI and EcoRI sites (5′ and 3′, respectively). The 15H and LMM cDNAs were subsequently cloned into pGEX-6P-1 (GE Lifesciences) as previously described [24], which adds a GST sequence and a PreScission protease site at the N-terminus, introducing the sequence GPLGS (derived from the expression vector) between GST and either LMM or the 15H construct. All constructs were sequenced to check for sequence fidelity. Mutant versions of the 15H constructs and the LMM constructs were then generated using the Quick Change-XL II Site Directed Mutagenesis Kit (Agilent).

### Expression and purification of LMM and 15H constructs

The WT and mutant constructs were transformed into *E. coli* Rosetta 2 (Novagen), a single colony was inoculated into a 5-ml starter culture and grown overnight before being added to 400 ml Terrific Broth (Sigma) and grown at 37 °C. When the OD_600_ reached ~ 0.8, cultures were induced with 0.5 mM IPTG. The 15H constructs were then expressed for 3 h at 37 °C, while LMM constructs were expressed at 20 °C overnight. Cells were harvested and washed in PBS, and the pellets stored at − 80 °C.

For protein purification, the pellets were resuspended in lysis buffer [PBS, 1 mM DTT, 1 mM EDTA, 200 μg/ml lysozyme, 0.1% Triton X-100, protease cocktail inhibitor tablet (Roche; pH 7.5)] for 30 min at room temperature on a roller. The lysates were sonicated on ice (6 cycles of 10 s on, 10 s off) and the cell debris was pelleted by centrifugation (3000*g*, 20 min). Proteins were purified by GST-tag affinity chromatography using Glutathione Sepharose 4B (GE Lifesciences).

For CD experiments, the GST was cleaved off the 15H constructs by a series of subsequent steps. First, cleared lysates were added to the equilibrated 5-ml polypropylene column (Qiagen) containing glutathione Sepharose 4B and washed with 5 column volumes of PBS, and the 15H protein was liberated from the column matrix by overnight incubation with PreScission Protease in cleavage buffer [50 mM Tris–HCl, 150 mM NaCl, 1 mM EDTA, 1 mM DTT (pH 7.5)] at 4 °C. The constructs were then further purified using anion exchange chromatography using a hiTrap 5 ml Q Sepharose column in 20 mM Tris–HCl (pH 8.0) and a gradient of 0.1 to 1 M NaCl.

The LMM constructs were purified using affinity chromatography in the same way as the 15H constructs. However, in this case, all buffers contained 500 mM NaCl to prevent LMM oligomerization. For EM studies, the GST tag was not cleaved away from LMM constructs, and the intact GST–LMM was eluted from the column by using 20 mM reduced glutathione. For CD experiments, LMM was diluted into 100 mM NaCl to allow for polymerization, followed by ultracentrifugation at 310,000*g* for 30 min. Protein was recovered by resuspending the pellets in high salt buffer [500 mM NaCl, 10 mM phosphate buffer (pH 7.5), 1 mM DTT], which solubilizes the protein.

To assess the purity of the protein (15H and LMM), fractions were collected, the protein content and purity were assessed by SDS-PAGE, and protein concentration was measured using the Bradford Assay (Sigma).

### CD

CD spectra were measured at 10 °C from 260 to 190 nm using an APP Chirascan CD spectropolarimeter. The CD buffer used for 15H constructs contained 50 mM NaCl, 1 mM DTT and 10 mM phosphate buffer (pH 7.4). The CD buffer used for LMM contained 500 mM NaCl, 1 mM DTT and 10 mM phosphate buffer (pH 7.4). The concentrations for all 15H and LMM constructs were 150–200 μg/ml. Scans at 10 °C were repeated twice, and a minimum of three experiments were performed. To calculate any significant difference, *t* tests of the 222-nm MRE value were used. An MRE value at 222 nm of 36,000 for a fully helical construct (100%) [31] was used to estimate the % helical content of the 15H constructs. Thermal melting measurements were taken at 1 °C increments from 10 to 85 °C, with 0.7 °C/min heating rate, in the same buffer.

### Gel filtration

A GE Healthcare Tricorn 10/20 column was packed with Superdex 75 resin and used at 20 °C. It was calibrated using the GE Healthcare gel filtration calibration kit, which comprises albumin (75 kDa), ovalbumin (43 kDa), carbonic anhydrase (29 kDa), ribonuclease A (13.7 kDa) and aprotinin (6.5 kDa). The elution profiles of the proteins of interest were obtained by injecting 400 μl of protein sample within a concentration range of 40–80 μM (monomer) in column buffer [150 mM NaCl, 10 mM sodium phosphate 1 mM DTT, 0.03% NaN_3_ (pH 7.4)] onto the column at a flow rate of 0.5 ml/min, using an AKTA system. The column exclusion volume was 6.3 ml (obtained using dextran blue). Elution of proteins was monitored by UV absorption at a wavelength of 220 nm.

### Solubility assays

WT and mutant GST–LMM proteins were dialyzed at 4 °C into 20 mM sodium phosphate, 500 mM NaCl and 1 mM DTT at pH 7.4. Each protein was then divided into five aliquots and diluted with 20 mM sodium phosphate, and 1 mM DTT (pH 7.4) to obtain the following NaCl concentrations: 100, 150, 200, 250 and 300 mM. Dilutions were performed to keep volume and protein concentration the same in each tube. Proteins were left on ice for 2 h for equilibration after which insoluble protein polymers were pelleted by ultra-centrifugation at 100,000*g* at 4 °C for 60 min using a TLA 100 rotor (Beckman Coulter®). Two 5-μl aliquots were removed from each supernatant for protein concentration quantification via μBCA assay.

### EM

GST–LMM was rapidly diluted into 150 mM NaCl in 10 mM phosphate buffer (pH 7.5) and then left on ice for 2 min to allow for filament formation. The GST–LMM final concentration was 0.3–0.5 μM. Ten microliters was then loaded onto a carbon-coated copper grid that had previously been irradiated under a UV lamp for 20 min [32]. The sample was washed three times in the dilution buffer and stained with 1% uranyl acetate. A Tecnai T12 microscope was used to image the grids and micrographs were recorded using a 2k × 2k Gatan CCD camera at a pixel size of 0.34 nm. Image measurements were performed using ImageJ software.

### Expression of eGFP–β-MHC in cardiomyocytes and myotubes

C2C12 myoblasts, used as a source for generating skeletal muscle myotubes in culture, were purchased from Public Health England culture collections. They were maintained in Dulbecco's minimum essential medium, with high glucose and Glutamax), supplemented with 20% FCS and 1% penicillin and streptomycin (diluted from stocks containing 10,000 U/ml). The differentiation medium was Dulbecco's minimum essential medium supplemented with 2% equine serum and 1% penicillin and streptomycin. The adenovirus expression vector, pDC315, was used to express the full-length heavy chain of *MYH7* (GenBank accession number M58018).

eGFP was fused to the N-terminus in the eGFP–MHC construct. Virus was generated and purified using Vivapure AdenoPACK 100 (Sartorius). The virus was titered and an MOI of 50–100 was used to infect cultured C2C12 myoblasts and isolated rat cardiomyocytes.

C2C12 myoblasts were seeded at the same cell density (1 × 10^5^ cells/ml) on coverslips coated with laminin-1 (Sigma). After 24-h infection with adenovirus, the growth medium was exchanged for differentiation medium and the cells were incubated at 37 °C for 5 days to allow for differentiation into skeletal muscle myotubes, before being fixed as described [34].

Isolated adult rat cardiomyocytes were prepared as described previously [24], [33]. Immediately after isolation, the cardiomyocytes were seeded onto laminin coated 13-mm diameter coverslips (50 μl at 50 μg/ml) at 1 × 10^5^ cells/ml and were infected with adenovirus on the day of isolation. Cells were then incubated for 24 h at 37 °C to allow for MHC expression, before being fixed with 2% formaldehyde for 20 min.

### Immunostaining and microscopy

Cells were stained using an antibody against the Z-line protein, α-actinin (A7811, Sigma), myosin heavy chain (A4.1025 antibody [22]) or an antibody against myomesin (Sigma). The secondary antibody used was anti-mouse Alexa Fluor 546 (Molecular Probes) and DAPI (Molecular Probes) was used to visualize the nucleus. For sarcomere organization analysis, images of cells were obtained using a Deltavision deconvolution microscope at high resolution (using the 100 × NA 1.4 objective). The same camera settings were used to capture all the images.

ImageJ was used to analyze sarcomeric incorporation of eGFP–MHC in skeletal myotubes. In ImageJ, lines of a fixed width were drawn along 4–5 sarcomeres in the same myofibril, and the intensity profile along the line was measured using the plot profile function. The data were combined using an Excel spreadsheet. Next, intensity profiles for each sarcomere along a profile for a single myofibril were aligned to the minimum value in the center of the sarcomere and used to generate an average plot for all the sarcomeres in that myofibril. The average values for each myofibril were then combined and averaged across myofibils, again aligning on the minimum value. Values for a minimum of 12 myofibrils were measured for each mutant and used to calculate the overall mean values and generate the profile plots.

Incorporation of eGFP–MHC in isolated adult rat cardiomyocytes was performed as described earlier [24]. Briefly, fixed cells were examined using an Olympus microscope, using the × 100, N.A. 1.3 oil objective, and scored for full or partial integration, or aggregated/no integration. At least 30 cells per construct were examined, and the experiment was repeated three times. Results were expressed as % full, partial or no integration (aggregated).

### Western blotting

Levels of eGFP–MHC expression in adult rat cardiomyocytes were compared to endogenous levels of MHC by SDS-PAGE gel analysis using a protocol that separates MHC isoforms in a minigel system [35]. Briefly, the separating gel contained 35% v/v glycerol, 9% w/*v* acrylamide–Bis (49:1), 230 mM Tris–HCl, 115 mM glycine (pH 8.8) and 0.4% w/v SDS, and the stacking gel contained 47% v/v glycerol, 6% w/v acrylamide–Bis (49:1), 110 mM Tris–HCl, 6 mM EDTA (pH 6.8) and 0.4% w/v SDS. Gels were stained, or proteins were transferred to nitrocellulose paper to perform Western blotting, using an antibody to MHC (A4.1025) or to eGFP (Abcam). These blots confirmed which band was endogenous and which the eGFP-tagged myosin in the stained gel. For myotubes, the protein samples were run on standard 7.5% SDS-PAGE gels, and expression levels of eGFP-tagged protein were assayed by Western blotting using the anti-eGFP antibody. Antibodies to α-tubulin (Biorad, MCA78G) were used on the same blots to test for equal loading of the protein samples. Blots were imaged with a LICOR C-digit blot scanner.

### MD simulations

Simulations using explicit solvent were performed using the CHARMM-36 force field parameters [36] with TIP3P water. A starting structure for the composite model of residues 1526–1689 within the LMM region of human β-MHC [25] was generously supplied by Professors Ivan Rayment and Qiang Cui. MD simulations were also performed on shorter segments of this same part of the β-MHC coiled coil for which experimental atomic structures are available [5]. For these, the N-terminal globular Xrcc4 moieties were removed using CHARMM-GUI [37]. Specifically, sequence 1562–1608 was taken from 5CJ4 (chain A: residues 1562–1615 and chain B: residues 1562–1608, [5]; sequence 1590–1657 was taken from 5CHX [5], and sequence 1631–1689 was taken from 5CJ0 [25]. Methylated lysine side chains (modified to facilitate crystallization in 5CJ4 and 5CHX) were all converted back to unmodified lysine. In all cases, N-termini were capped with acetyl groups and C-termini were capped with *N*-methylamide for both chains. Atomic structures equivalent to the WT composite model were also made for the mutants A1603P and K1617del. The A1603P mutant structure was generated from the WT structure by replacing the Ala residue with the most probable Pro rotamer (for chain A: *χ*_1_ − 32.4, *χ*_2_ 42.1 and for chain B: *χ*_1_ 21.0, χ_2_ − 23.3) indicated by the Structure Edit function within Chimera [38]. Generation of a starting structure for the K1617del mutant is described below.

Starting structures were energy minimized for 1000 steepest decent steps in vacuum using CHARMM [39]. Using VMD [40], a 1.5-nm surround of water molecules and Na^+^ and Cl^−^ ions were then added to neutralize the construct and give a NaCl concentration of ~ 150 mM. A 10,000-step minimization, 0–300 K heating protocol and short pre-equilibration run (100,000 steps) were performed using NAMD. Data are taken from simulation runs lasting at least 360 ns using NAMD at 300 K. The timestep used was 2 fs and trajectory frames were recorded every 5000 steps. The first 10 ns of each run was removed prior to analysis to avoid starting structure bias. For the shorter coiled coil sections, simulations ran for 275 ns, with frames recorded every 500 steps.

Simulation trajectories were analyzed using Wordom [41]. The helicity of both chains was calculated using backbone dihedral angles and the method previously described [36]. RMSD values for all Cα atoms were calculated with respect to the initial structure, while RMSF values on a per residue basis were calculated with respect to an averaged structure. Structures were clustered based on the Cα RMSD values using a cutoff value of 8 Å [25]. The mean ± SD distances between helices (*D*_com_) values were calculated using a moving window to include the positions of seven Cα (3 N-terminal and 3 C-terminal to the marked residue in each chain). Similarly, average heptad lengths along each helix were calculated using a moving window average of Cα positions in seven (*i*) residues and their (*i* + 7) residue partners in the sequence. The inter-heptad angles were measured between Cα atoms in residues at sequence positions (*i* – 7), *i*, and (*i* + 7). The program Naccess was used to show which residues make up the hydrophobic core of the protein ([42] and Hubbard, S. J.; Thornton, J. M. Naccess; Department of Biochemistry and Molecular Biology, University College London. http://www.bioinf.manchester.ac.uk/naccess/). The relative solvent accessibility (probe size 1.4 Å) of side-chain atoms within each residue from simulation snapshots output at 10-ps intervals was calculated and averaged (Cα atoms were included as part of the side chain).

We validated our simulations of the composite model, which used a different MD force field (from Ref. [23]) and explicit (rather than implicit) solvent, by comparing the *D*_com_ along the length of our WT composite model with those of shorter sections of the same part of the β-MHC coiled coil that are available direct from X-ray crystal structures (Fig. S3A). Away from the termini of the shorter sections, which would be expected to be more variable, there is excellent agreement. This good structural agreement is again shown on comparison of the residue side chain solvent accessibility (Fig. S3B), which shows that the same pattern of core and exposed residues is retained across models. The individual helix parameters (local heptad length and inter-heptad angle) are again well matched across simulations (Fig. S3C and S3D). They highlight some conserved areas of asymmetry between the two helices, most notably through the inter-heptad angle around residue 1665 and to a lesser extent near residue 1580. Thus, despite using a different underlying force field, in terms of *D*_com_, RMSF, RMSD, and the associated clustering results, the composite WT model in our hands behaves in a very similar manner to that reported [25].

Producing a model for the K1617del mutant was more challenging than for the proline mutant, since simply deleting K1617 from the WT model and rebonding the chains across the gap produces a break in the coiled coil structure that would not occur during formation of the mutant coiled coil *in vivo*. We therefore built *ab initio* a non-canonical coiled coil WT and K1617del model using the program BEAMMOTIFCC [43]. These models used non-canonical structures for the skip residue and for the deletion site. The initial WT model was built using the following values: 3.617 residues per turn of helix, an axial translation per residue of 1.495 Å, and a relative rotation of the two helical strands of 210°. The major helical radius used was 4.9 Å, which was the average major helical radius calculated along the coiled coil from simulations of the composite PDB model. The smoothing parameter *b* was varied to find a value that resulted in simulation average properties that best matched those from simulating the composite PDB model (*b* = 0.03 was the optimum). The structure is defined in the program by a pattern of “motifs” that link equivalent residues in the sequence; the motif for a canonical coiled coil being 7-amino-acid residues. To accommodate the skip residue, E1582, a 29-residue motif replaced four 7-residue motifs between F1565 and V1594. We validated the method by comparing this 164-residue WT model with the composite model. Starting simulations from our *ab initio* model gave rise to average properties that all matched very well those found for simulations initiated from the composite model, showing that the method copes well with skip residues (Fig. S4).

We then used the same approach to build a K1617del coiled-coil model with a smooth all-helix structure from which to initiate simulations. To build a non-canonical K1617del model with a continuous helical structure, in addition to the skip motif modification, a 27-residue motif replaced four canonical 7-residues motifs between L1601 and L1629 (WT numbering is used here and throughout for K1617del). The BEAMMOTIFCC program was modified in order to implement the 27-residue motif, which is defined to contain 8 helical turns (like in the WT four 7-residue motifs that it replaced) to ensure a left-handed coiled coil (refer to the *N* value in [43] for details). To test whether the skip residue at 1582 and the deletion at 1617 can cancel each other out, a second K1617del model was built that used a canonical coiled-coil motif throughout this region.

BEAMMOTIFCC provides a backbone structure for the coiled-coil model. Side-chain atoms were added to the model using SCWRL4 [44]. N-termini were again capped with acetyl groups and C-termini were capped with N-methylamide. Simulations were run as for the composite model with an additional minimization and equilibration run with restrained backbone atoms to allow for side-chain only equilibration (10,000-step minimization, 0–300 K heating protocol and 100,000 step pre-equilibration performed using NAMD) prior to the all atom minimization.

## Appendix A Supplementary data

Supplementary figures Image 1

## References

[1] McLachlan A.D., Karn J. (1982). Periodic charge distributions in the myosin rod amino acid sequence match cross-bridge spacings in muscle. Nature.

[2] Craig R., Woodhead J.L. (2006). Structure and function of myosin filaments. Curr. Opin. Struct. Biol..

[3] Hu Z., Taylor D.W., Reedy M.K., Edwards R.J., Taylor K.A. (2016). Structure of myosin filaments from relaxed Lethocerus flight muscle by cryo-EM at 6 A resolution. Sci. Adv..

[4] Squire J.M. (1973). General model of myosin filament structure. 3. Molecular packing arrangements in myosin filaments. J. Mol. Biol..

[5] Taylor K.C., Buvoli M., Korkmaz E.N., Buvoli A., Zheng Y., Heinze N.T., Cui Q., Leinwand L.A., Rayment I. (2015). Skip residues modulate the structural properties of the myosin rod and guide thick filament assembly. Proc. Natl. Acad. Sci. U. S. A..

[6] Colegrave M., Peckham M. (2014). Structural implications of beta-cardiac myosin heavy chain mutations in human disease. Anat. Rec. (Hoboken).

[7] Chou P.Y., Fasman G.D. (1978). Empirical predictions of protein conformation. Annu. Rev. Biochem..

[8] Meredith C., Herrmann R., Parry C., Liyanage K., Dye D.E., Durling H.J., Duff R.M., Beckman K., de Visser M., van der Graaff M.M., Hedera P., Fink J.K., Petty E.M., Lamont P., Fabian V., Bridges L., Voit T., Mastaglia F.L., Laing N.G. (2004). Mutations in the slow skeletal muscle fiber myosin heavy chain gene (MYH7) cause laing early-onset distal myopathy (MPD1). Am. J. Hum. Genet..

[9] Lamont P., Wallefeld W., Davis M., Udd B., Laing N. (2011). Clinical utility gene card for: Laing distal myopathy. Eur. J. Hum. Genet..

[10] Barlow D.J., Thornton J.M. (1988). Helix geometry in proteins. J. Mol. Biol..

[11] Parry D.A., Fraser R.D., Squire J.M. (2008). Fifty years of coiled-coils and alpha-helical bundles: a close relationship between sequence and structure. J. Struct. Biol..

[12] Srikakulam R., Liu L., Winkelmann D.A. (2008). Unc45b forms a cytosolic complex with Hsp90 and targets the unfolded myosin motor domain. PLoS One.

[13] Armel T.Z., Leinwand L.A. (2010). A mutation in the beta-myosin rod associated with hypertrophic cardiomyopathy has an unexpected molecular phenotype. Biochem. Biophys. Res. Commun..

[14] Armel T.Z., Leinwand L.A. (2010). Mutations at the same amino acid in myosin that cause either skeletal or cardiac myopathy have distinct molecular phenotypes. J. Mol. Cell. Cardiol..

[15] Craig R.W., Knight P.J., Harris J.R. (1983). Myosin molecules, thick filaments and the actin-myosin complex. Electron Microscopy of Proteins.

[16] Billington N., Wang A., Mao J., Adelstein R.S., Sellers J.R. (2013). Characterization of three full-length human nonmuscle myosin II paralogs. J. Biol. Chem..

[17] Zhou N.E., Kay C.M., Hodges R.S. (1994). The role of interhelical ionic interactions in controlling protein folding and stability. De novo designed synthetic two-stranded alpha-helical coiled-coils. J. Mol. Biol..

[18] Ward R., Bennett P.M. (1989). Paracrystals of myosin rod. J. Muscle Res. Cell Motil..

[19] Huxley H.E. (1963). Electron microscope studies on the structure of natural and synthetic protein filaments from striated muscle. J. Mol. Biol..

[20] Obermann W.M., Gautel M., Weber K., Furst D.O. (1997). Molecular structure of the sarcomeric M band: mapping of titin and myosin binding domains in myomesin and the identification of a potential regulatory phosphorylation site in myomesin. EMBO J..

[21] Obermann W.M., van der Ven P.F., Steiner F., Weber K., Furst D.O. (1998). Mapping of a myosin-binding domain and a regulatory phosphorylation site in M-protein, a structural protein of the sarcomeric M band. Mol. Biol. Cell.

[22] Maggs A.M., Taylor-Harris P., Peckham M., Hughes S.M. (2000). Evidence for differential post-translational modifications of slow myosin heavy chain during murine skeletal muscle development. J. Muscle Res. Cell Motil..

[23] Buvoli M., Buvoli A., Leinwand L.A. (2012). Effects of pathogenic proline mutations on myosin assembly. J. Mol. Biol..

[24] Wolny M., Colegrave M., Colman L., White E., Knight P.J., Peckham M. (2013). Cardiomyopathy mutations in the tail of beta-cardiac myosin modify the coiled-coil structure and affect integration into thick filaments in muscle sarcomeres in adult cardiomyocytes. J. Biol. Chem..

[25] Korkmaz E.N., Taylor K.C., Andreas M.P., Ajay G., Heinze N.T., Cui Q., Rayment I. (2016). A composite approach towards a complete model of the myosin rod. Proteins.

[26] Fiorillo C., Astrea G., Savarese M., Cassandrini D., Brisca G., Trucco F., Pedemonte M., Trovato R., Ruggiero L., Vercelli L., D'Amico A., Tasca G., Pane M., Fanin M., Bello L., Broda P., Musumeci O., Rodolico C., Messina S., Vita G.L., Sframeli M., Gibertini S., Morandi L., Mora M., Maggi L., Petrucci A., Massa R., Grandis M., Toscano A., Pegoraro E., Mercuri E., Bertini E., Mongini T., Santoro L., Nigro V., Minetti C., Santorelli F.M., Bruno C., Italian Network on Congenital, Myopathies (2016). MYH7-related myopathies: clinical, histopathological and imaging findings in a cohort of Italian patients. Orphanet J. Rare Dis..

[27] Armel T.Z., Leinwand L.A. (2009). Mutations in the beta-myosin rod cause myosin storage myopathy via multiple mechanisms. Proc. Natl. Acad. Sci. U. S. A..

[28] Lamont P.J., Wallefeld W., Hilton-Jones D., Udd B., Argov Z., Barboi A.C., Bonneman C., Boycott K.M., Bushby K., Connolly A.M., Davies N., Beggs A.H., Cox G.F., Dastgir J., DeChene E.T., Gooding R., Jungbluth H., Muelas N., Palmio J., Penttila S., Schmedding E., Suominen T., Straub V., Staples C., Van den Bergh P.Y., Vilchez J.J., Wagner K.R., Wheeler P.G., Wraige E., Laing N.G. (2014). Novel mutations widen the phenotypic spectrum of slow skeletal/beta-cardiac myosin (MYH7) distal myopathy. Hum. Mutat..

[29] Papageorgopoulos C., Caldwell K., Schweingrubber H., Neese R.A., Shackleton C.H., Hellerstein M. (2002). Measuring synthesis rates of muscle creatine kinase and myosin with stable isotopes and mass spectrometry. Anal. Biochem..

[30] Miller G., Maycock J., White E., Peckham M., Calaghan S. (2003). Heterologous expression of wild-type and mutant beta-cardiac myosin changes the contractile kinetics of cultured mouse myotubes. J. Physiol..

[31] Greenfield N., Fasman G.D. (1969). Computed circular dichroism spectra for the evaluation of protein conformation. Biochemistry.

[32] Burgess S.A., Walker M.L., Thirumurugan K., Trinick J., Knight P.J. (2004). Use of negative stain and single-particle image processing to explore dynamic properties of flexible macromolecules. J. Struct. Biol..

[33] Leach R.N., Desai J.C., Orchard C.H. (2005). Effect of cytoskeleton disruptors on L-type Ca channel distribution in rat ventricular myocytes. Cell Calcium.

[34] Parker F., White K., Phillips S., Peckham M. (2016). Promoting differentiation of cultured myoblasts using biomimetic surfaces that present alpha-laminin-2 peptides. Cytotechnology.

[35] Picard B., Barboiron C., Chadeyron D., Jurie C. (2011). Protocol for high-resolution electrophoresis separation of myosin heavy chain isoforms in bovine skeletal muscle. Electrophoresis.

[36] Best R.B., Zhu X., Shim J., Lopes P.E., Mittal J., Feig M., Mackerell A.D. (2012). Optimization of the additive CHARMM all-atom protein force field targeting improved sampling of the backbone phi, psi and side-chain chi(1) and chi(2) dihedral angles. J. Chem. Theory Comput..

[37] Jo S., Kim T., Iyer V.G., Im W. (2008). CHARMM-GUI: a web-based graphical user interface for CHARMM. J. Comput. Chem..

[38] Pettersen E.F., Goddard T.D., Huang C.C., Couch G.S., Greenblatt D.M., Meng E.C., Ferrin T.E. (2004). UCSF Chimera—a visualization system for exploratory research and analysis. J. Comput. Chem..

[39] Brooks B.R., Brooks C.L., Mackerell A.D., Nilsson L., Petrella R.J., Roux B., Won Y., Archontis G., Bartels C., Boresch S., Caflisch A., Caves L., Cui Q., Dinner A.R., Feig M., Fischer S., Gao J., Hodoscek M., Im W., Kuczera K., Lazaridis T., Ma J., Ovchinnikov V., Paci E., Pastor R.W., Post C.B., Pu J.Z., Schaefer M., Tidor B., Venable R.M., Woodcock H.L., Wu X., Yang W., York D.M., Karplus M. (2009). CHARMM: the biomolecular simulation program. J. Comput. Chem..

[40] Humphrey W., Dalke A., Schulten K. (1996). VMD: visual molecular dynamics. J. Mol. Graph..

[41] Seeber M., Felline A., Raimondi F., Muff S., Friedman R., Rao F., Caflisch A., Fanelli F. (2011). Wordom: a user-friendly program for the analysis of molecular structures, trajectories, and free energy surfaces. J. Comput. Chem..

[42] Lee B., Richards F.M. (1971). The interpretation of protein structures: estimation of static accessibility. J. Mol. Biol..

[43] Offer G., Hicks M.R., Woolfson D.N. (2002). Generalized Crick equations for modeling noncanonical coiled coils. J. Struct. Biol..

[44] Krivov G.G., Shapovalov M.V., Dunbrack R.L. (2009). Improved prediction of protein side-chain conformations with SCWRL4. Proteins.

