# Supplementary material for: A1603P and K1617del, Mutations in β-Cardiac Myosin Heavy Chain that Cause Laing Early-Onset Distal Myopathy, Affect Secondary Structure and Filament Formation In Vitro and In Vivo
Source: J Mol Biol. 2018 May 11;430(10):1459–78. doi: 10.1016/j.jmb.2018.04.006 (PMC5958240; doi:10.1016/j.jmb.2018.04.006)
Supplement: Supplementary file 1 — Supplementary figures [file mmc1.docx]

**Supplementary data for:**

**A1603P and K1617del, Mutations in β-Cardiac Myosin Heavy Chain that Cause Laing Early-Onset Distal Myopathy, Affect Secondary Structure and Filament Formation *In Vitro* and *In Vivo***

Francine Parker, Matthew Batchelor, Marcin Wolny, Ruth Hughes, Peter J. Knight and Michelle Peckham

**
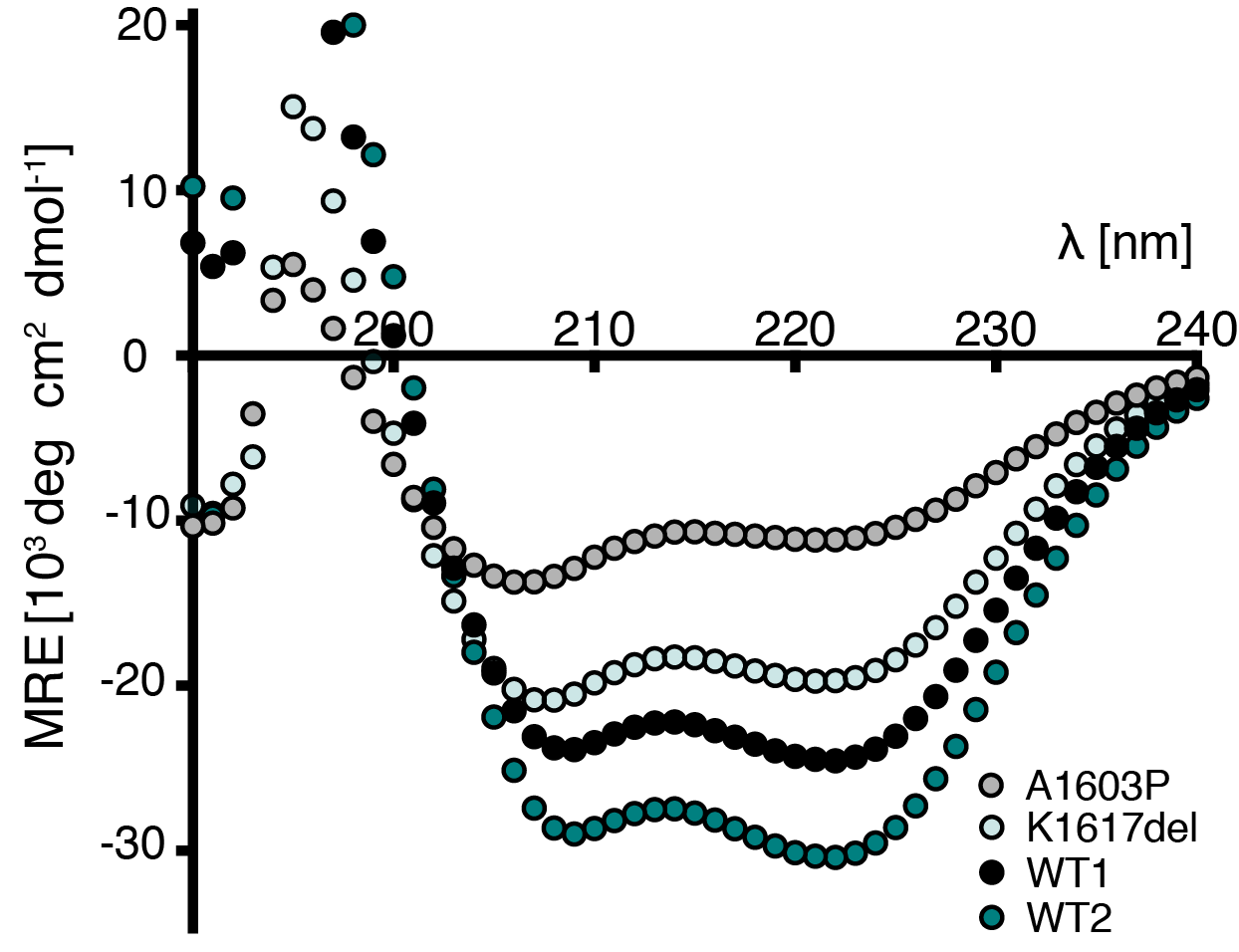
**

**Fig. S1**. CD of 15H constructs in 500 mM NaCl. These experiments were performed as described for CD at low salt (Fig. 2). Data below 200 nm are compromised by the high absorption of 500 mM Cl^–^ ions. The mutations had an impact on WT 15H helicity that is similar to that observed at low salt.


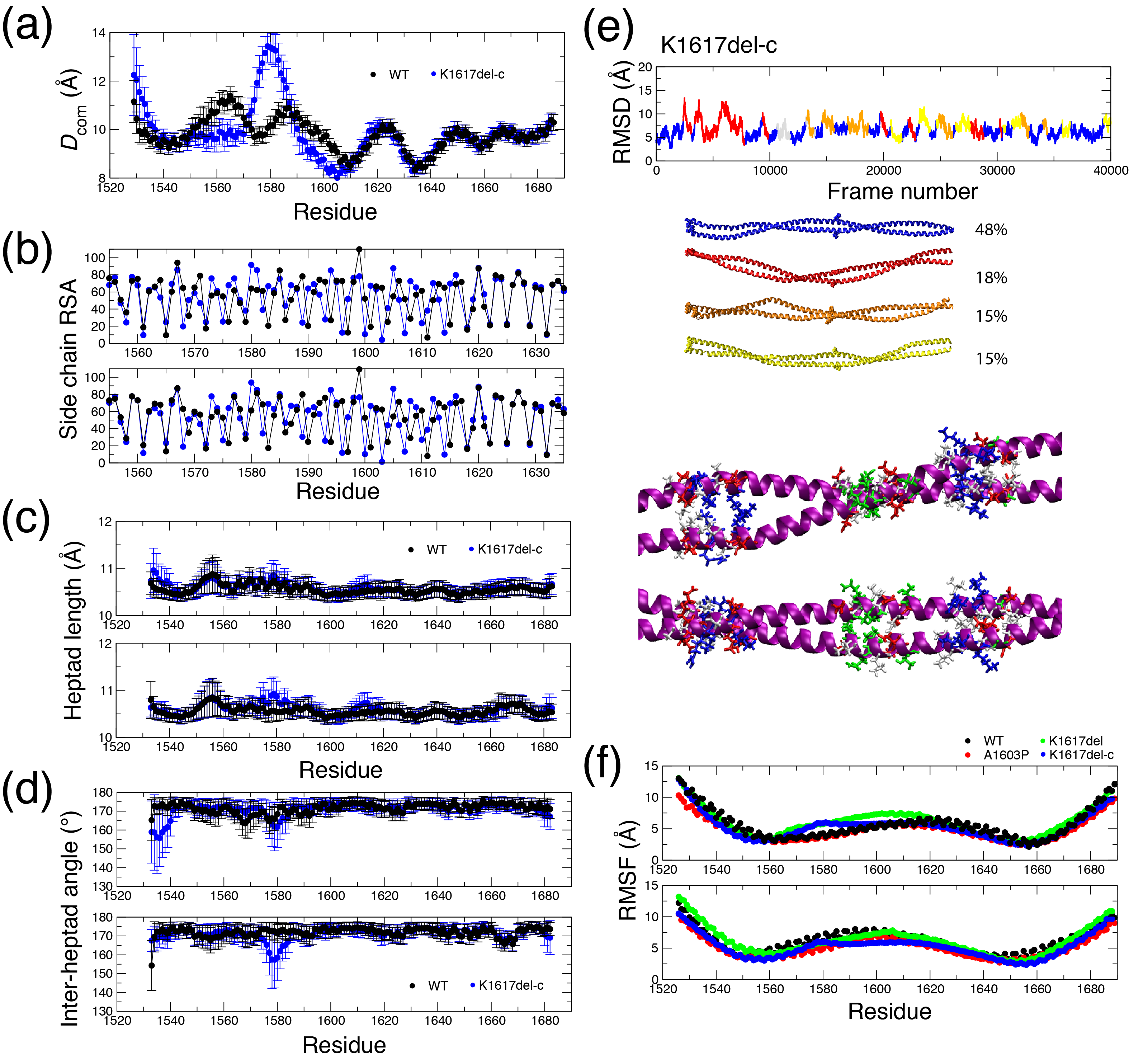


**Fig. S2**. Results from simulations of an alternative K1617del model (K1617del-c) generated using a canonical coiled coil sequence pattern. Comparisons are made with the composite WT model simulations. (a) Local distances between the helices (*D*_com_) along the coiled coil. Error bars represent standard deviation during the simulation. A large increase in distance between the helices is observed near the skip residue site at E1582. (b) Side chain relative solvent accessibility (RSA) for chain A (upper) and chain B (lower). Error bars are omitted in (b) for clarity. (c) Heptad length along the sequence for chain A (upper) and chain B (lower). (d) Inter-heptad angle along the sequence for chain A (upper) and chain B (lower). The plot also includes data for A1603P and the non-canonical K1617del model. (e) Root mean squared deviation (RMSD) for all Cα atoms compared to the initial structure for K1617del-c. The plot is divided by colour into the different structure clusters. Example structures for the most heavily populated clusters (>10%) are shown beneath. K1616 is shown in spacefill. The cluster populations as a percentage of all trajectory snapshot structures are indicated. Beneath those are snapshot images showing core residue regions in a representative structure from the most heavily populated cluster (blue 48%). The lower image is the upper structure rotated around the major axis of the coiled coil. The regions with side chains drawn are those most different from WT. The region 1570–1580 near the skip residue (E1582) (left) has a run of several charged residues in core (“*a*” or “*d*”) heptad positions: K1575 (“*d*”), K1579 (“*a*”) and E1582 (“*d*”). The region 1595–1605 (centre) has a run of several residues with small side chains in core heptad positions: S1596 (“*d*”), S1600 (“*a*”), A1603 (“*d*”) and S1607 (“*a*”). The region 1611–1621 surrounding the deletion site is also shown (right). (f) Root mean square fluctuation (RMSF) of Cα atoms about an averaged structure for chain A (upper) and chain B (lower). Results for WT, A1603P and both K1617del models are shown.

**Fig. S3**. Results from simulations of the composite model compared to those for the coiled coil sections of PDB structures 5CJ4, 5CHX and 5CJ0. (a) Distance between the helices (*D*_com_). (b) Side chain relative solvent accessibility (RSA) for chain A (upper) and chain B (lower). (c) Heptad length along the sequence for chain A (upper) and chain B (lower). (d) Inter-heptad angle along the sequence for chain A (upper) and chain B (lower). Note the chain names for 5CJ0 were swapped to better match the asymmetry observed.


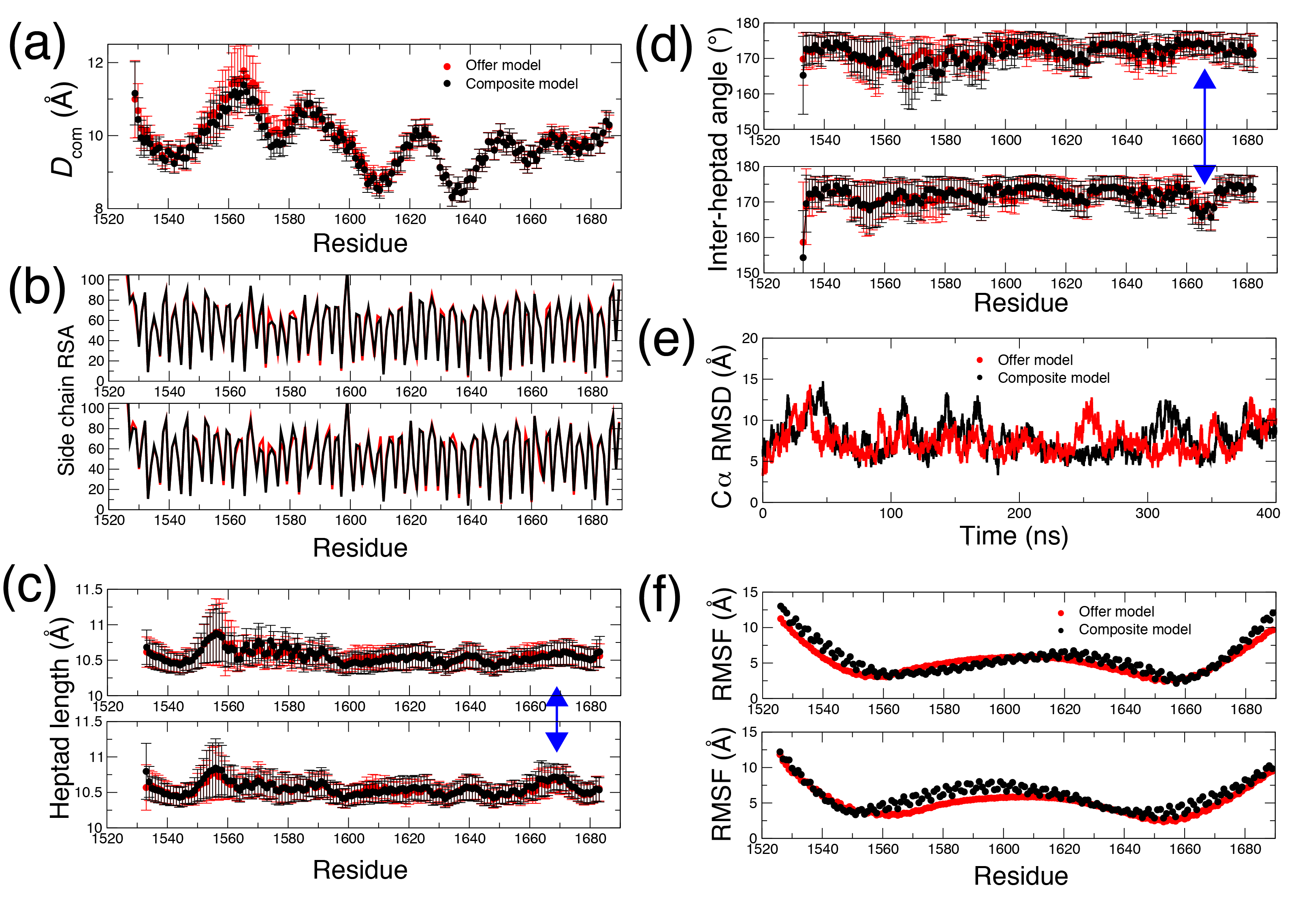


**Fig. S4**. Results from simulations of the WT model generated using the Offer *et al*. method compared to the composite model. (a) Distance between the helices (*D*_com_). (b) Side chain relative solvent accessibility (RSA) for chain A (upper) and chain B (lower). (c) Heptad length along the sequence for chain A (upper) and chain B (lower). (d) Inter-heptad angle along the sequence for chain A (upper) and chain B (lower). The blue arrows in part (c) and (d) highlight regions of asymmetry between chains that are produced in both models. (e) Root-mean-square deviation (RMSD) of Cα atoms in structures compared to the initial composite model structure in both cases. (f) Root mean square fluctuation (RMSF) of Cα atoms about an averaged structure for chain A (upper) and chain B (lower).
